# Mapping national information and communication technology (ICT) infrastructure to the requirements of potential digital health interventions in low- and middle-income countries

**DOI:** 10.7189/jogh.12.04094

**Published:** 2022-12-29

**Authors:** Chi Yan Hui, Adina Abdulla, Zakiuddin Ahmed, Himanshi Goel, G M Monsur Habib, Toh Teck Hock, Parisa Khandakr, Hana Mahmood, Animesh Nautiyal, Mulya Nurmansyah, Shweta Panwar, Rutuja Patil, Fedri Ruluwedrata Rinawan, Hani Salim, Ashish Satav, Jitendra Nandkumar Shah, Akshita Shukla, Chowdhury Zabir Hossain Tanim, Dominique Balharry, Hilary Pinnock, the RESPIRE Group

**Affiliations:** 1 NIHR Global Health Research Unit on Respiratory Health (RESPIRE), Usher Institute, University of Edinburgh, UK; 2 Department of Primary Care Medicine, Faculty of Medicine, University Malaya, Kuala Lumpur, Malaysia; 3 Riphah Institute of Healthcare Improvement & Safety and Secretary, Islamabad, Pakistan; 4 Centre for Technology Alternatives for Rural Areas (CTARA), Indian Institute of Technology Bombay, Mumbai, India; 5 Bangladesh Primary Care Respiratory Society (BPCRS), Khulna, Bangladesh; 6 Clinical Research Centre, Sibu Hospital, Ministry of Health Malaysia, Kuala Lumpur, Malaysia; 7 Medinova Medical Services Ltd, Dhaka, Bangladesh; 8 Neoventive Solutions, Islamabad, Pakistan; 9 Indian Institute of Technology Bombay, Mumbai, India; 10 Departmentof Public Health, Faculty of Medicine, Universitas Padjadjaran, Bandung, West Java, Indonesia; 11 Vadu Rural Health Program, King Edward Memorial Hospital Research Centre Pune, India; 12 Department of Family Medicine, Faculty of Medicine and Health Sciences, University Putra Malaysia, Kuala Lumpur, Malaysia; 13 MAHAN Trust, Mahatma Gandhi Tribal Hospital, Maharashtra, India; 14 Indian Institute of Technology Bombay, Mumbai, India; 15 Department of Physical Medicine and Rehabilitation, Sylhet MAG Osmani Medical College Hospital, Sylhet, Bangladesh

## Abstract

**Background:**

Digital health can support health care in low- and middle-income countries (LMICs) by overcoming problems of distance, poor infrastructure and the need to provide community practitioners with specialist support. We used five RESPIRE countries as exemplars (Bangladesh, India, Indonesia, Malaysia, Pakistan) to identify the digital health solutions that are valuable in their local setting, worked together with local clinicians and researchers to explore digital health policy, electricity/ICT infrastructure, and socio-cultural factors influencing users’ ability to access, adopt and utilise digital health.

**Methods:**

We adopted the Joanna Briggs Institute’s scoping review protocol and followed the Cochrane Rapid Review method to accelerate the review process, using the Implementation and Operation of Mobile Health projects framework and The Extended Technology Acceptance Model of Mobile Telephony to categorise the results. We conducted the review in four stages: (1) establishing value, (2) identifying digital health policy, (3) searching for evidence of infrastructure, design, and end-user adoption, (4) local input to interpret relevance and adoption factors. We used open-source national/international statistics such as the World Health Organization, International Telecommunication Union, Groupe Speciale Mobile, and local news/articles/government statistics to scope the current status, and systematically searched five databases for locally relevant exemplars.

**Results:**

We found 118 studies (2015-2021) and 114 supplementary online news articles and national statistics. Digital health policy was available in all countries, but scarce skilled labour, lack of legislation/interoperability support, and interrupted electricity and internet services were limitations. Older patients, women and those living in rural areas were least likely to have access to ICT infrastructure. Renewable energy has potential in enabling digital health care. Low usage mobile data and voice service packages are relatively affordable options for mHealth in the five countries.

**Conclusions:**

Effective implementation of digital health technologies requires a supportive policy, stable electricity infrastructures, affordable mobile internet service, and good understanding of the socio-economic context in order to tailor the intervention such that it functional, accessible, feasible, user-friendly and trusted by the target users. We suggest a checklist of contextual factors that developers of digital health initiatives in LMICs should consider at an early stage in the development process.

Low- and middle-income countries (LMICs) face many challenges providing health care for their populations. Digital health solutions can overcome the problems of distance, poor transport infrastructure, limited medical provision in rural areas and the need to provide rural practitioners with specialist support [1]. Recent telehealth examples from the National Institute for Health and Care Research (NIHR) Global Health Research Unit on Respiratory Health (RESPIRE) include digital auscultation to improve diagnosis of paediatric pneumonia [2], computational frameworks to interpret chest x-rays [3], home-based pulmonary rehabilitation [4], mHealth support for Community Health Workers [5,6], remote teleconsultation facilities (Micro-Health Centres) [7] mobile-based interventions for adults with asthma with limited health literacy [8]^,^ and blended learning for professional [9,10]. However, poor mobile signal coverage [11] and limited availability of mobile/smart devices [12] hinder the application of digital health in rural and/or deprived communities.

There is a need when planning digital health projects to understand the local information and communications technology (ICT) infrastructure to ensure interventions are developed and delivered in such a way that they are accessible to the majority of the population. This review, commissioned by RESPIRE, aimed to scope the current ICT context in member countries and to develop a practical approach that could be applied to future projects proposed by partners in South/Southeast Asia.

We aimed to identify and describe the evidence available in open-source data and existing literature regarding implementation of digital health applications in five exemplar RESPIRE LMICs (Bangladesh, India, Indonesia, Malaysia, Pakistan). Our questions were:

1. What digital health policy, electricity and ICT infrastructures are available to support digital health?
2. What socio-cultural factors influence access to the infrastructures, and users’ ability to adopt and utilise digital health?

## METHODS

The review was conducted from May to December 2021, by colleagues from the RESPIRE partner countries in an iterative process to ensure that findings would address local needs.

We adopted the Joanna Briggs Institute’s scoping review protocol [13], and followed Cochrane Rapid Review methodology to accelerate the review process [14]. Clinicians and researchers from the RESPIRE countries formed the review team enabling us to frame the questions to ensure the knowledge generated would address the practical factors relevant to the digital health projects they are planning, as well as the general issues that need to be considered in a successful technology initiative in an LMIC. We use PRISMA (scoping reviews extension) to report the data. PROSPERO does not accept scoping review registrations, but the search strategy, inclusion criteria, exclusion criteria, and analysis plan were specified a priori in a protocol that was reviewed by the RESPIRE group.

### Over-arching framework

**Figure 1:**
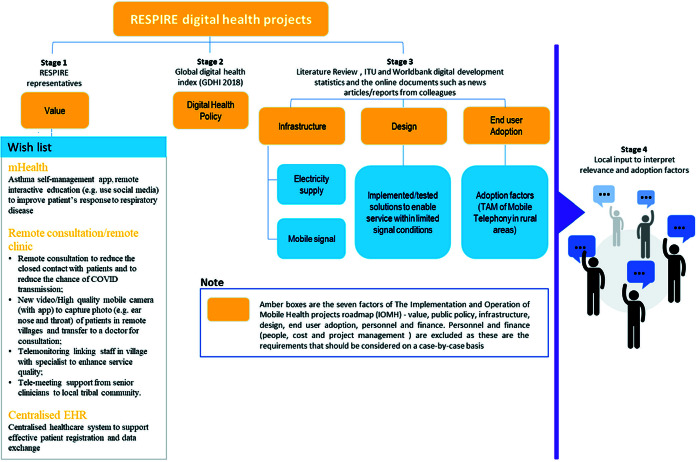
Review flowchart using the Implementation and Operation of Mobile Health projects roadmap.

The Implementation and Operation of Mobile Health projects roadmap (IOMH-roadmap) [15] summarises the policy, infrastructure and end-user factors to be considered for a successful digital health implementation (Question 1). The seven factors that contribute to the current status and enabling environment, are value, public policy, infrastructure, design, end-user adoption, personnel and finances. We investigated five factors in this review, excluding personnel and finance as these are the requirements of people, costs and project management related to specific digital health projects and hence should be considered on a case-by-case basis with guidance such as the World Health Organization (WHO) Digital implementation investment guide 2020 (DIIG) [16]. **Figure 1** illustrates how we shaped our review around this framework. We followed a four-stage process to collect data on the five factors from the IOMH-roadmap.

### Stage 1. Establishing value

The local RESPIRE clinicians and researchers defined the digital health applications they thought would be valuable in their countries (we called this the “wish list”). We used the technologies in the “wish list” to inform the search terms and eligibility criteria in Stage 3.

### Stage 2. Identifying digital health public policy

Digital policy provides insight into government support and potential available funding to implement digital health applications in the countries. We searched the countries in the Global Digital Health Index (GDHI) which “scores” the local leadership and governance, strategy and investment, legislation, policy and compliance, workforce, standards and interoperability, infrastructure and the digital health services and applications to provide an understanding of local policy[17].

### Stage 3. Searching for evidence of infrastructure, design, and end-user adoption

Infrastructure refers to electricity and mobile signal coverage required to implement digital health. Adoption is influenced by the user-friendliness of the design, the end user’s attitude towards the technology and the socio-cultural context (eg, barriers related to gender, age, and affordability of technology). We sought evidence of research at local community level and the latest statistics of technology ownership and signal penetration at country level. The procedure is described below with additional details in **Table 1**.

**Table 1:** Scoping review search strategy

	Inclusion and exclusion criteria, data range and sources of searches
**Definitions used in this scoping review**	**IT infrastructure is the hardware and software to support data or electricity transmission** Examples in this review of –Hardware are personal digital assistant (PDA), call machine, 2G-5G mobile phones, sensors, computer, electrical socket point and signal tower.Software can refer to a computer or mobile application and its architectures such as applications on its interoperability layer (use of API/SKD) and presentation layer (app interface design).
**Initial questions**	(Primary) What IT infrastructure is available to support digital health in different countries/regions?(Secondary) What socio-cultural factors influence the access to IT infrastructure, and ability to adopt and utilise digital health?
**Population**	Stakeholders of digital health such as users (patients and clinicians), technology developers, health /technology service providers and researchers.
**Intervention**	Any IT infrastructure that could support a digital health intervention.
**Settings**	Any health care setting in the five exemplar countries.
**Study focus**	All studies, clinical and technology systematic/scoping reviews, best practice framework, and grey literature (eg, WHO and GSMA reports), open-source IT infrastructure statistics with themes that relate to digital health for respiratory health in India, Indonesia, Malaysia, Pakistan or Bangladesh.
**Languages**	All
**Date range**	January 1, 2015 to June 18, 2021. The date limit of 2015 ensured we only included recently completed infrastructures. The typical implementation lifecycle of ICT infrastructure is three to five years.
**Databases**	Open-source telecommunication databases and mobile signal/electricity grid map (ITU, WHO, International energy agency (https://www.iea.org/data-and-statistics), World bank and include any open sources suggested by colleagues/ found to be useful /in literature or the statistics.Web of Science Core Collection and ISI Proceedings (SCI-EXPANDE; SSC; A&HCI; CPCI-S; CPCI-SSH; BKCI-S; BKCI-SSH), IEEE Xplore Digital Library, Compendex, Inspec & Knovel, Scopus (5 databases in total).
**Search terms**	mhealth OR Remote consultation telemonitoring/remote clinic OR centralised electronic health record OR the specific technologies that could be used to implement the projects in the “wish list”.
**Exclusion criteria**	Exclude in the first phase: data out of range, proposing new technology/ tool only (geographic information system (GIS)/Zigbee flooding system). Exclusion criteria: 1. Study that was not in Bangladesh, India, Indonesia, Malaysia and Pakistan. 2. Study did not use on-site data to evaluate the local public infrastructure services (eg, author’s opinions on existing infrastructure without analysis). 3. Study on new digital health or ICT infrastructures without implementation and on-site evaluation. (eg, author’s opinions on new technology). 4. Study on electricity demand, internet service and mobile consumption modelling without providing the reasons for services disturbance (eg, demand prediction model). 5. Study on digital adoption assessment methodologies or reporting on co-design workshops without providing technical/legislation/socio-cultural factors that influence the access to existing IT infrastructure, and the users’ ability to adopt and utilise existing ICT infrastructure. We used specific criteria to exclude renewable energy simulation studies. 6. Renewable energy review before Jan 2021 Author opinion to provide suggestions to renewable energy policy. 7. Study not reporting potential energy generation or cost for the system such as Cost per energy (COE). 8. Study for specific commercial building or robot or smart city planning. 9. Study without site evaluation or justifications on the choices of the simulation parameters.

We searched five databases and the International Telecommunication Union (ITU) digital development statistics, to identify the existing infrastructures, their design and end-user adoption factors relevant to the valued technology that we identified in stage 1 [18]. Search terms were formed from the RESPIRE partners’ “wish-list” (see **Table 1** for the search terms and searched databases). We included studies (implementation projects, case studies, technical reports and systematic/scoping reviews) that assessed performance and user experience of locally implemented infrastructures including renewable technology that could generate electricity to support digital health applications with a date range of January 1, 2015 to June 18, 2021.

### Screening and study selection

Title and abstracts were screened by one reviewer (CyH). The full text of all potentially eligible studies were retrieved and assessed against the inclusion criteria (**Table 1**) by CyH and checked by second reviewers from the RESPIRE countries (TZ, RP, SP, AS, AH, HG, HS, HM and HP). Initial agreement was 88%, but after discussion we achieved 100% agreement.

### Data extraction

Due to the resources and time limitations, one reviewer (CyH) extracted data using a piloted extraction sheet under heading characteristics of the included studies (publication year, study methods and conclusion). Study design, intervention, and outcomes were extracted where appropriate (some papers were reviews or performance evaluations). We extracted all the data from the study as reported, and did not contact study authors for additional information. CyH presented the data extraction results in the group meetings inviting feedback on the coherence of the findings with the real-life scenario in the local areas.

### Stage 4. Local input to find supplementary data, and interpret relevance

RESPIRE colleagues suggested additional publications and strategies to identify local information not addressed in the (English) published literature and the GDHI [17]. We used the IOMS-roadmap to categorise the studies according to the factors addressed: infrastructure (electricity, mobile signal, renewable energy), end user adoption and design.

Custom searches were performed using a Google search engine to find supplementary data (for example: local news web links) about the issue of electricity that was lacking of addressing in the literatures but were found important from the RESPIRE colleagues in the real life implementation Local search terms such as the “power outage”, “electricity issue”, “100% electrification”, “un-electrified villages” were used Thruuu data scraper was used to extract data from the Google search [19].

As a scoping review, we did not assess the quality of the included studies. Our aim was to identify the breadth and sources of data available, and we therefore provide a descriptive synthesis of the authors’ conclusions in terms of the intervention and population characteristics, technologies and study outcomes.

### The roles of the reviewers

The first reviewer (CyH) has experience in working with multi-cultural teams in the UK and Asia, with background in engineering and public health studies. Second reviewers (TZ from Bangladesh; HG, RP, SP and AS from India; HS from Malaysia; MN from Indonesia, HM from Pakistan), and other RESPIRE colleagues are local researchers or clinicians, who are fluent in English and their local languages. They duplicated the selection at full text stage. HP is a senior clinical academic from the UK who oversaw the process and acted as an advisor and, as necessary, an arbiter.

### Narrative data synthesis

Question 1: We synthesised the conclusions of the heterogeneous studies, categorised by the IOMH-roadmap infrastructure (electricity, mobile signal, renewable energy), end user adoption and design.

Question 2: We plotted effective end-users’ adoption factors (eg, perceived usefulness and ease of use [20,21]), infrastructure and study characteristics on a harvest plot. Over and above individual preferences, various socio-cultural factors (eg, availability of technology gender inequalities) influence what is available and possible in the context of LMICs. The adoption factors were categorised by the “micro-factors” (personal image, mobility, cost of service and handset, social influence, perceived health hazard, service quality, transparency of service offerings, mass media, ease of service availability) of the Extended Technology Acceptance Model (TAM) of Mobile Telephony [22].

### Mapping the review data with the real-life scenario

We used the International Telecommunication Union (ITU) and the World Bank open sources statistics to triangulate the data extracted from the studies [18,23]. The RESPIRE teams in the five countries commented on the review data, and identified any gaps or discrepancies between those data and the real-life scenario that they observed in everyday practice. We developed a descriptive summary of the ITU and World Bank data with all the review findings.

## RESULTS

### Characteristics of the included studies and online open sources (all stages)

**Figure 2:**
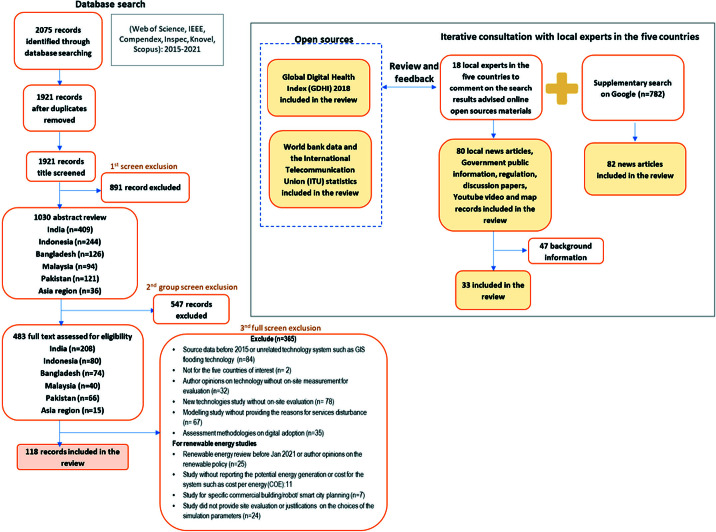
PRISMA flowchart.

The identified papers and open-source online records, the screening process, and the final number of studies and records are detailed in the PRISMA flowchart (**Figure 2**).

**Table 2** shows the data we included in the review in the various stages. 10 colleagues (clinicians and clinical/technology researchers) from Bangladesh, India, Indonesia, Malaysia and Pakistan completed a survey on the valued digital health applications in their countries and the potential day-to-day challenges to form a “wish list”. Open source data such as the Global Digital Health Index, the World Bank data and the International Telecommunication Union (ITU) statistics provided national data (excluding India) [17,18,23]. The database search retrieved 118 studies, and local colleagues advised customised google searches that identified 82 local news articles about the quality of electricity and internet services that they had observed was an important issue but was not addressed in the statistics and studies. An additional 33 online records such as local news articles, Government public information, regulation, discussion papers, YouTube videos and map records, supplemented missing data.

**Table 2:** Process, timeline and data included in the review stages

Stages in the search plan	Sources	May 2021	June 2021	July 2021	Aug 2021	Sept 2021	Oct 2021	Nov 2021	Dec 2021	Records included
1. Value	Survey to local RESPIRE teams (Bangladesh, India, Indonesia, Malaysia and Pakistan)	**Stage 1**								10 local experts and their teams in the five countries replied to the survey
2.Digital health public policy	Global Digital Health Index (GDHI) 2018		**Stage 2**							GDHI of 4 countries Bangladesh, Indonesia, Malaysia, Pakistan
3.Infrastructure, end user, design	Database search (Web of Science, IEEE, Compendex, Inspec, Knovel, Scopus): 2015-2021			**Stage 3**						118 studies from 5 databases (2015-2021)
	World bank data and the International Telecommunication Union (ITU) statistics									Access to electricity and internet ICT, price and affordable cost
4. Feedback from local RESPIRE colleagues	Google search to explore further issues after feedback from the group							**Stage 4**		82 online records (Appendix 1)
On-going iterative discussions	Feedback collected from group meetings	**On-going iterative discussions**								33 online records (Appendix 2)

### The included studies

We included 118 studies in the review (Appendix 3 in the **Online Supplementary Document**)and, following the IOMH-roadmap (**Figure 1**), grouped them into six categories (electricity (n = 15), renewable energy (n = 47), mobile signal coverage (n = 20), design (n = 17) and end-user adoption (n = 19)). Appendix 3 in the **Online Supplementary Document** provides details of the included studies. Of the 118 studies, most were from India (39%, 46/118), others were from Indonesia (21%, 25/118), Pakistan (17%, 20/118), Malaysia (12%, 14/118) and Bangladesh (11%, 13/118). India also contributed most of the papers in the renewable energy category (45%, 21/47).

### 1: Value

The “wish-list” of digital health care from the five RESPIRE countries was broad and encompassed features of m-Health, telehealth and centralised electronic health records (EHRs) (**Table 3**). m-Health includes digital health applications that use (web/standalone) mobile apps as a medium to support patients’ health care [24]. Telehealth is the use of telecommunications and virtual technology to deliver health care outside traditional health care facilities [25]. The most valued digital health applications were information provision (for patients and professional development), mapping service availability, remote consulting, remote monitoring of patients’ condition, remote tracking of field staff (eg, when conducting home visit to patients), open centralised health care system and air pollution monitoring.

**Table 3:** Wish list: Valued digital health applications and observed challenging in the five countries (India, Indonesia, Malaysia, Bangladesh and Pakistan)

Wish list in the five countries (India, Indonesia, Malaysia, Bangladesh and Pakistan)	
**Pakistan**	• A service availability mapping system; diagnosis and referral app for under five pneumonia; a SMS chatbot in Roman Urdu about respiratory information; interactive mobile game to spread correct health care information and encourage self-reporting of data; remote health information for patients.
**Malaysia**	• Asthma self-management app¸app for clinical guideline information and updates.
**Indonesia**	• An open (FHIR) centralised health care system.
**India**	• Telehealth platforms with implementation research to encourage the adoption and use; air pollution mapping; remote consultations between village communities and hospitals; digital examinations: high quality mobile camera to capture specialist images (eg, of ear nose, and throat, video laryngoscopy, and transfer to doctor/technicians for consultation; telemonitoring to support field staff – enabling joint consultant consultation; remote interactive education (eg, use social media) to improve patient’s response to respiratory disease; tele-meeting support with senior clinicians for the local tribal community members.
**Bangladesh**	• Exploratory study (with online questionnaire) on physical activities or pulmonary rehabilitation.
**Observed challenges in the five countries** (India, Indonesia, Malaysia, Bangladesh and Pakistan)	
**Policy**	• Interoperability: different providers and systems in primary/sary practices, lack of standardised protocol makes it difficult to share medical information and make joint decisions on patient management; regulation: lack of government regulations to use/store health data to drive a seamless centralised health care; no clear legal framework on how to use technology for health care delivery (liability issues); government: no clear development roadmap on digital health, diverse local policies (**Pakistan**); human resources: lack of skilled manpower in community health projects, and limited technology support.
**Infrastructure**	• Poor mobile coverage in urban and remote area; limited/unstable internet coverage especially in remote areas; unstable electricity supply (seasonal peaks (**Pakistan**), rainy season (**India**)), lack of electrical charging points; lack of equipment (eg, digital stethoscope) and computer hardware (dependents on importing (**Pakistan**); no real time dashboard for hospital colleagues to monitor telemedicine devices; lack of software resources and poor maintenance to update the software; multiple mobile suppliers in one area, need two sim cards to access mobile signal (**India**); multiple mobile OS/device resolution makes user interface design difficult; telecommunication infrastructure is monopolised by government-link company, challenging to improve service (eg, too focused on fibre deployment as opposed to affordable and faster internet wiring to wider community); technology companies want profitable technology (**Pakistan**).
**Social cultural adoption and utilisation**	• Low mobile phone ownership. Households may have one smart phone shared between family; affordability of internet plan/access to internet; low technology literacy in both clinicians and patients; low health literacy in patients; lack of confidence in data protection and effectiveness of technology; language: multilingual community want technology in their own language (**Malaysia**); lack of awareness of available digital health services, and unsure about the health benefits, digital divide.

**Table 3** also lists the potential challenges to implementing digital health applications identified by the colleagues in their local settings. Challenges such as poor/unstable electricity supply and mobile signal coverage, unaffordability of mobile phone ownership/access to internet, were applicable in all the countries, especially in rural areas. No clear legal framework on use of technology for health care access and delivery was a concern because of lack of clarity who (the clinician, the health care organisation, patient, technology manufacturer) were liable if the application went wrong. Lack of skilled manpower and hardware/software equipment challenged deployment of their wish-list of digital health solutions.

### 2: Digital health policy

**Figure 3:**
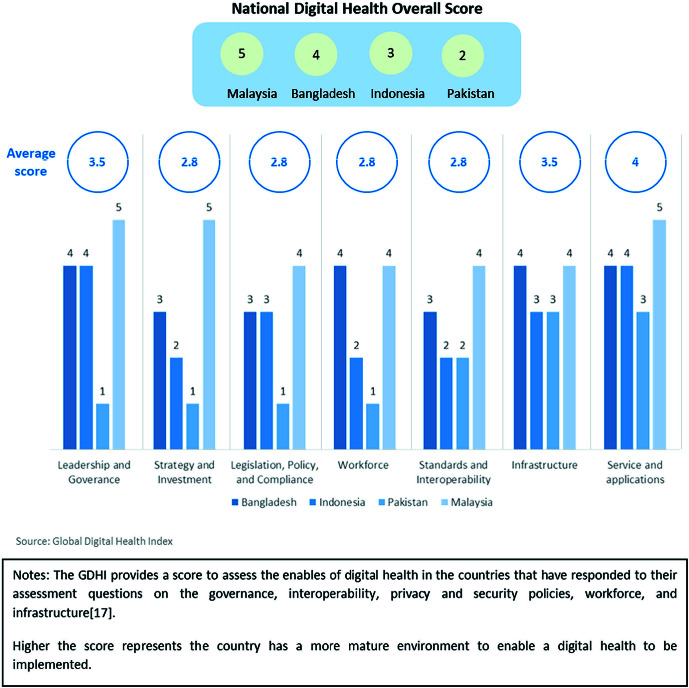
Global Digital Health Index (GDHI) 2018 for Bangladesh, Indonesia, Malaysia, and Pakistan (no scores available for India). The GDHI provides a score to assess the enables of digital health in the countries that have responded to their assessment questions on the governance, interoperability, privacy and security policies, workforce, and infrastructure [17]. Higher the score represents the country has a more mature environment to enable a digital health to be implemented.

The GDHI, which benchmarked self-reported scores across seven domains for Bangladesh, Indonesia, Malaysia, and Pakistan in 2018 (**Figure 3**), described 50%-75% of the population in Malaysia, Bangladesh and Indonesia, and 25%-50% of the population in Pakistan as “supported by digital health systems”. In Bangladesh and Malaysia, the public health care facilities were tagged to allow Geographic Information Systems to map their location. Secured patient registries were available for more than 75% of population in Indonesia and Malaysia; in Bangladesh and Pakistan, registries were available for less than a quarter of the population and irregular maintenance limited usage.

Although the governments of Bangladesh, Indonesia and Malaysia had prioritised digital heath in relevant national plans and had strategies for upgrading hardware and software for health care purposes, legislation and standards for interoperability were less well addressed. Lack of professionals with expertise in health informatics and minimal training for clinicians and community health workers were identified as limitations in Indonesia and Pakistan. Pakistan did not have a coordinating body for digital health, and scored relatively poorly in categories related to leadership, governance, investment strategy, legislation, and policy.

The GDHI does not include scores for India [17], however, the Indian government’s Ayushman Bharat Digital Mission, describes a “citizen-centric” National Digital Health Blueprint and the key building blocks for India’s Digital Health Ecosystem. The Blueprint defines data standards/regulations, describes plans for unique patient digital IDs, registries of private and public health facilities and health care professionals, and increased digital access for the population [26-28]. There is less clarity within the implementation plan on developing expertise in health informatics and on provision of training for clinicians and community health workers [25].

### 3: Infrastructure, design, and end user adoption

#### Infrastructure: Electricity

Electricity is essential for deployment and operation of a digital health service. Mobile phones need to be charged to deliver a mhealth service, remote clinics need lighting and computers to support video-consultations and diagnostic testing, electricity is needed to transmit mobile signals.

In 2019, the percentage of the population with access to electricity was 100% (or nearly) in urban areas of all RESPIRE countries. However (apart from Malaysia) rural areas were less well served, especially in Bangladesh and Pakistan where the proportion of the rural population with access to electricity was 89% and 59% respectively (Appendix 4 in the **Online Supplementary Document**) [21]. In reality, however, the affordability, reliability, and duration of access to electricity remains an issue [29] (also see Appendix 1 and Appendix 2 in the **Online Supplementary Document**). For example, in the rural areas of India, 50% of households experienced power cuts for 8 hours/d [30]. In Pakistan, electricity shortage results in power outages for 8-10 hours in urban areas while rural areas face load-shedding up to 18 hours/d [31]. Flooding in the rainy season in Malaysia caused electricity outages [32].

Renewable energy could potentially provide 24-hour/d electricity in rural areas, either on-grid (as an alternative electricity supply when the grid is lost) or off-grid (as an electricity source for areas without grid coverage). ZigBee PRO protocol (with together with sensors and embedded system) could be a cost-effective option to support full integration of intelligent Distributed Renewable Generation (DRG) and small scale Building-Integrated Photovoltaic (BIPV) [33]. In the techno-economic studies of hybrid renewable energy that we identified across the five countries (Appendix 3 in the **Online Supplementary Document**), 47 studies reported the potential to generate 6.55-30 kWh/d off-grid electricity in rural areas, that could support digital health applications such as mHealth and a remote clinic as well as enable basic mobile signal transmission (see Appendix 3 in the in the **Online Supplementary Document**, 72-118 renewable energy category). By reducing energy insecurity, such schemes have multiple other socio-economic benefits for local households [34], mitigating electricity costs, greenhouse gas emissions, and (in Bangladesh) improving political trust [35].

Renewable energy policies exist in all five countries, reflecting their governments’ intention to develop sustainable energy in rural areas and in response to climate change. However, critics predict delays as plans are complex or vague (eg, in Pakistan [36]) or financial and governance procedures are unclear (eg, in Bangladesh [37]). There are significant logistical challenges deploying these alternative power supplies in rural or remote tribal areas, such as lack of local skilled labour for installing equipment and problem solving, procedural delays in approving deployment, lack of incentives, market and business environments to sustain off-grid electricity [32].

#### Infrastructure: Mobile signal coverage and adoption cost

**Figure 4:**
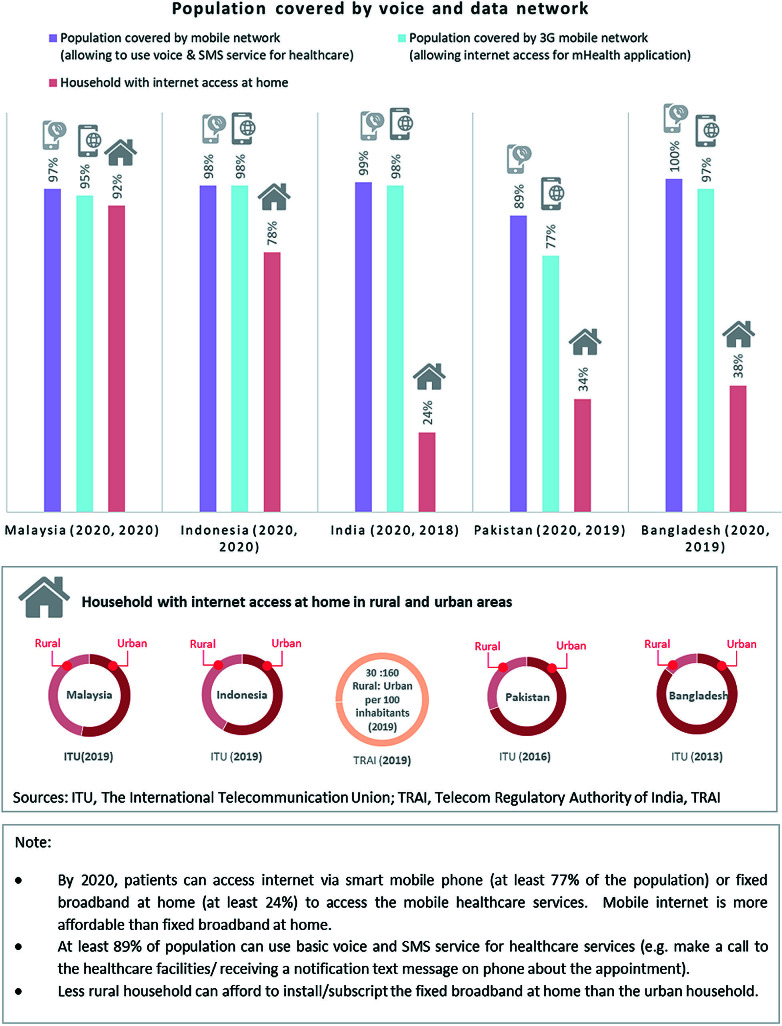
Network coverage 2020. By 2020, patients can access internet via smart mobile phone (at least 77% of the population) or fixed broadband at home (at least 24%) to access the mobile health care services. Mobile internet is more affordable than fixed broadband at home. At least 89% of population can use basic voice and SMS service for health care services (eg, make a call to the health care facilities/ receiving a notification text message on phone about the appointment). Less rural household can afford to install/subscript the fixed broadband at home than the urban household.

**Figure 4** and Appendix 4 in the **Online Supplementary Document** provide details of the mobile signal data. By 2020, mobile and data networks covered at least 77% of the population in the five countries with greater coverage in urban than in rural areas [38-40]. In contrast, the proportion of households with home broadband access to the internet is low in India (24% in 2019), Pakistan (34% in 2019) and Bangladesh (38% in 2019). This low uptake is correlated with the cost of fixed broadband packages which in all the countries is above the “2% of monthly Gross National Income per capita” (GNIpc) affordability threshold. This is particularly true in Indonesia and Pakistan in which fixed broadband costs an estimated 11% of GNIpc (compared to 3.2% in India; 2.6% in Bangladesh; 2.2% in Malaysia). In contrast, mobile data and voice services are a relatively affordable option for mHealth in all the countries.

Poor/unstable signal quality was a concern [41-46] despite innovative strategies to support mobile signal in rural areas. Published studies, observation of local colleagues and the local news articles identify factors such as obstruction from buildings/trees, distance to transmitter, high use and limited capacity that lead to signal disturbance (**Table 4**). Practical considerations which act as barriers include unprofitable business models (eg, in Pakistan [87]), and multiple service providers.

**Table 4:** Mobile signal: signal disturbance factors, proposed technologies and exemplar digital health intervention summary

Signal disturbance factors	**The building, trees, network density and heavy rains can cause reflection, diffraction and scattering of the mobile signal [** **47** **-** **50** **]; long distances between transmitter to receiver in the rural villages decreases the signal received by the users [** **51** **]; multiple service providers, (for example in India) such that users need to subscribe to two sim cards in a mobile phone to maintain a seamless signal coverage [** **52** **]; poor indoor coverage where signals cannot penetrate (for example inside shopping malls) [** **53** **]; network characteristics. 4G networks perform much better than 3G networks (for example in regions of Malaysia) [** **54** **]; unexpected high use of internet can exceed the capacity of the infrastructure (for example during the COVID pandemic) [** **55** **]; loss of internet for political (for example in Indonesia) [** **56** **], internet service providers (for example in Pakistan) [** **57** **] or other organisational reasons.**
**Range of proposed technologies to provide mobile signal in rural areas**	Implementing 5G [58-62], utilising multi-operator core network to enable spectrum sharing [63], WebRTC API for real-time streaming [64]; High Throughput Satellite (HTS) to co-channel [65]; spectrum of DVB and GSM band [66]; utilising the existing TV bands to build a frugal 5G architecture [67-69].
**Exemplar digital health interventions**	**Remote clinic for consultation and diagnosis**: Despite limited internet access, in rural villages a remote digital clinic can enable specialist consultations and transfer diagnostic images to professionals in the hospitals for real-time advice. In India, teleconsultations used a specially designed ambulance, equipped to transmit 2G(voice) mobile data signals with a camera, VSAT broadband network, SMS/MMS, and 3G/4G internet antennae [70-73]. Of the four solutions, the VSAT broadband network and SMS/MMS were successfully implemented in 108 villages throughout India [74]. Raspberry Pi have been used in Telecytology, Tele-pathology system, Tele-EyeCare Service System to transfer images from areas with limited mobile network coverage to distant specialist centres for example in Indonesia and Bangladesh [74-76]. **Pregnancy monitoring system:** Three studies reported the mobile app monitoring tools deployed in India and Pakistan to monitor pregnancy [77-79]. The Daksh system monitored intra partum care [79]; the Continuum of Care mhealth platform was used for pregnancy registration and monitoring care [80], and a mhealth app in Pakistan managed the clinical workforce and provision of health care equipment [81]. **Healthcare facilities app**: A mobile app in Malaysia provided information about local facilities with maps, health care information, cost estimation, and online survey feedback [81]. **ICT in community pharmacies**: ICT features such as electronic health records, electronic payment systems, accounting and logistics software, barcode reading system for medicines, online stores, as well as systems for communication with patients (Whatsapp, SMS) were implemented in pharmacies in Malaysia. The accounting and logistics software was the most used feature [80].** Centralised electronic health record (EHR**): Five studies in India and Pakistan implemented proof-of-concept EHR systems in hospital and primary health centre with limited resources [82-86]. Major challenges were the lack of interoperable systems; logistical issues (unstable internet connections, lack of eHealth skills); staff perceptions that the system was time-consuming, differences in the native languages, gender imbalances and lack of national standardisation, security and legislative framework.

### Design and tested solutions

We identified 17 studies reporting digital health solutions that provided exemplar designs of the initiatives that RESPIRE colleagues had included on their “wish list” (**Table 4**). Examples included remote clinics for consultation and diagnosis, pregnancy monitoring system, centralised EHR, ICT in community pharmacies, though there were challenges such as lack of interoperable systems; unstable internet connections; and lack of security and legislative frameworks [82-86].

### End users’ adoption

**Figure 5:**
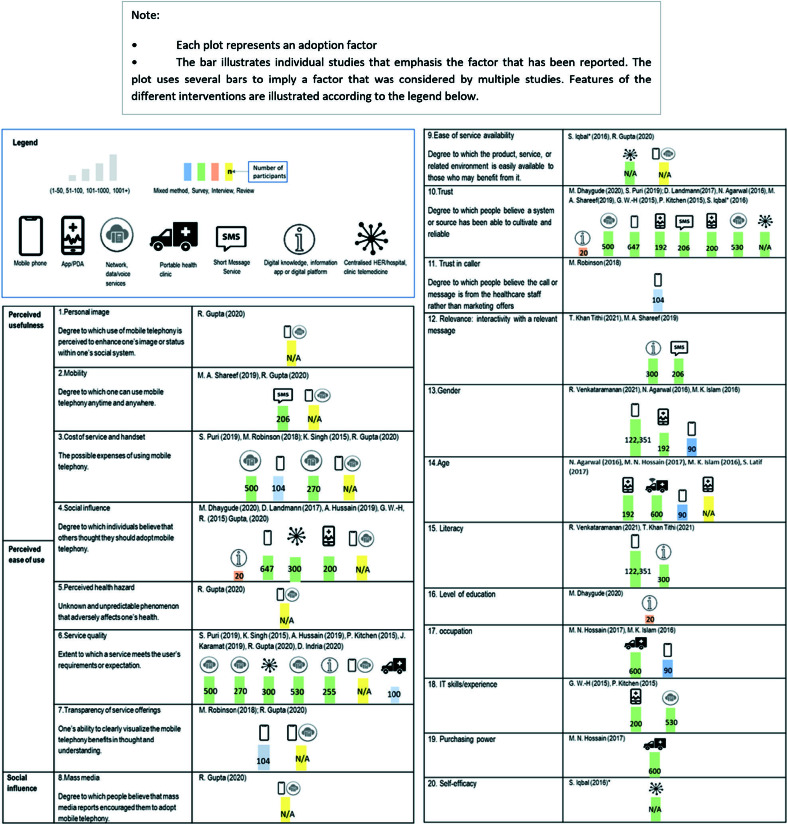
Harvest plots illustrating use of digital interventions related to adoption factors. Each plot represents an adoption factor. The bar illustrates individual studies that emphasis the factor that has been reported. The plot uses several bars to imply a factor that was considered by multiple studies. Features of the different interventions are illustrated according to the legend below.

We included 19 studies that reported users’ adoption of digital health care (Appendix 3 in the **Online Supplementary Document**), reporting multiple factors that affected usage. Trust was emphasised as influencing adoption: both trust in the intervention (the degree to which the participant believed the digital intervention was accurate or reliable), and trust in the caller (degree to which people believed the call or message was genuine (as opposed to marketing) [45,46,87-92]. Other factors included relevance of the information, gender, age, literacy, occupation, ICT skills/experience, and belief in ability to use the technology (the Harvest plot, **Figure 5**).

### 4. Local input to interpret relevance and adoption factors

We identified a significant gender gap in mobile ownership and use of internet in the five countries which is illustrated in Appendix 4 in the **Online Supplementary Document** [18,93,94]. Fewer women owned a mobile phone and used the internet in all the countries, though the gender gap was only 3% in Malaysia in 2020. Pakistan had the widest gap in mobile ownership (33% in 2020); India, Bangladesh and Pakistan had a wide gender gap in internet use (India:42% in 2020; Bangladesh: 38% in 2019; and Pakistan: 38% in 2017).

## DISCUSSION

### Summary of findings

RESPIRE colleagues from Bangladesh, India, Indonesia, Malaysia and Pakistan wished to implement a wide range of mhealth, telehealth and centralised EHR applications, but several practical barriers exist, challenging the deployment in their local areas. We investigated the national policy, infrastructure, design, and end user adoption to understand the challenges. Digital health policies were available in all the countries, but often lacked the backing of legislation and regulations governing standardisation and interoperability, and with limited strategies for ensuring a skilled workforce and training for clinicians in using digital technologies in routine practice. While almost all the population could access electricity, this was often unstable especially in rural areas making it difficult to achieve a reliable remote clinical service. Renewable energy may offer solutions for the future. Mobile internet coverage has increased over recent years and in urban (but not rural) areas is almost universal, but the cost of fixed broadband makes this unaffordable in the low-income countries. Substantial gender disparities in internet access challenge deployment of equitable digital health solutions. There are innovative studies from the five countries using technology that could be deployed to deliver the projects on the ”wish-lists” of the partners. Apart from ease of use and the perceived usefulness of the technology, users’ trust in the accuracy and reliability of the service was crucial to engaging users in the digital services.

### Strength and limitations

Our review scoped the current policy, infrastructure and enabling environment that form the context for the digital health solutions valued by RESPIRE colleagues. Our findings are specific to their South/South East Asian countries, though the approach and the open access sources used are likely to be applicable to other LMIC countries (and potentially remote/low-resource settings in high income countries) as considerations of policy, infrastructure, design and user-adoption are universally relevant. Our focus was on the context, so we did not consider the characteristics of the individual digital health project (thus we identified national workforce/costs data from the global digital heath index but not the specific requirements for people, costs and project management) which are inherent to planning a novel intervention and can be informed by WHO toolkits [16].

We searched for the most recent data available, but in some cases, these were a few years out of date. Accelerated by the COVID-19 pandemic, patients are increasingly motivated to adopt digital options for their health care and technology adoption status such as the mobile phone ownership and internet access gap could change rapidly [18,95-100]. Planning a new digital health intervention involves checking the open access sources that we have identified to gather the most up-to-date information.

There are some methodological limitations. Due to resources and time constraints, we were unable to duplicate the full selection and data extraction processes, though the single researcher (CyH) erred on the side of caution to reduce the risk of rejecting a useful publication. RESPIRE colleagues reviewed the full text screening for publications from their countries and contributed to operationalising the inclusion and exclusion criteria. Whilst this is a limitation for a systematic scoping review, it most likely represents the real-world scenario in a team developing a novel digital health intervention. Different colleagues (researchers/academics with different backgrounds) were involved in the five countries which might have led to different approaches, though the main researcher was common to all sites and we had a number of video-conference meetings at which views were exchanged to facilitate standardisation. Although we acknowledge this diversity between countries is a limitation, we believe this collaboration was also a strength that enabled this complex multinational project to be conducted. The multi-disciplinary background of the reviewer team provided a broad spectrum of opinions to be included in the scoping process and to facilitate a balanced interpretation.

In line with scoping review methodology, we did not assess quality and were reliant on the accuracy and completeness of the original authors’ descriptions of their results and conclusions. There were missing data in the open source statistics, though we used studies and the local news articles to supplement the missing information. Finally we will have missed emerging technologies that were not indexed in the databases.

### Interpretation in relation to the published digital health guidelines and literature

Assessing the ‘enabling environment’, including the current status of ICT infrastructure (power supply and internet network), the readiness of the national/local digital health policy (leadership & governance, strategy and investment, legislation, human resources, standards and interoperability, services and applications) and the factors that determine uptake (including the risk of increasing inequities) form the crucial context when designing a novel digital health solution. In this review, we provide a high-level review of these contextual issues for five South/Southeast Asian countries as a practical exemplar of applying the WHO DIIG advice [16] and the framework to improve the health service delivery [101]. We conclude that an assessment of these contextual issues should proceed in parallel (or even precede) developing specific plans. These are summarised in a checklist (**Table 5**).

**Table 5:** Checklist of contextual factors

Checklist of contextual factors that developers of digital health initiatives in LMICs should consider at an early stage in the development process	
Policy, legislation and standards	Be aware of the legislative context (and also the lack of regulation and standardisation) for digital health and ensure the solution complies with requirements – and also how lack of regulation will be managed.
Infrastructure	Identify national statistics on access to electricity and the internet, but also explore the reality which, especially in rural areas maybe unstable supply or “patchy” coverage. Consider implementing a strategy that enables the digital health solution to work “offline” and/or on battery mode. Consider renewable energy as an alternative source of electricity and using technologies such as Raspberry Pi, SMS, VSAT broadband network in areas with limited access to the internet.
Interoperability	Explore policies for interoperability, and ensure the digital health intervention is open and able to connect with the centralised health care system (if/when available).
Workforce	Consider the skilled workforce situation and allow adequate resources to address any shortfall. On-going technological support will be necessary to support the deployment and training for existing clinicians to operate the technologies.
Adoption costs	Understand the local costing structure for landline internet compared to low usage mobile data and voice service packages as the latter may be the only affordable option in LMICs and the technology needs to be designed accordingly.
Digital inequalities	Social norms and gender disparities in accessing mobile and internet, digital literacy and online safety are barriers for woman to access technologies. Understand the local context and adapt technology requirements to avoid increasing inequalities for women (and other disadvantaged groups: elderly, deprived communities, minority groups, poorly literate).
Encouraging adoption features	In addition to the typical factors of age, gender, and literacy, consider the key factors of perceived usefulness; perceived ease of use, service quality and the perceived trust in the technology when designing the features of the intervention. End users co-design will help ensure the intervention responds to the user’s needs.

We highlight a number of gaps in the policy context, infrastructure and workforce which echoes the findings of Leonard et al.’s systematic review on barriers and facilitators to implementing evidence-based interventions in LMICs [102]. In discussion with RESPIRE colleagues we extended our investigation to include gender imbalance, and the news items that exposed the variable reality behind headline statistics, especially in rural areas. United States Agency for International Development (USAID) further highlights the on-going financial investment needed to cover the cost of the technology replacement because of the theft, loss or damage and expected device life-span [103].

## CONCLUSION

Effective implementation of digital health technologies requires a supportive policy, stable electricity infrastructures, affordable mobile internet service, and good understanding of the socio-economic context in order to tailor the intervention such that it functional, accessible, feasible, user-friendly and trusted by the target users. Using digital health initiatives planned in five South/Southeast Asian countries as exemplars, we have searched open access sources and published literature to explore the context in discussion with local colleagues. Learning from our experience and findings we suggest a checklist of contextual factors that developers of digital health initiatives in LMICs should consider at an early stage in the development process.

## Additional material


Online Supplementary Document
