# Supplementary material for: Mapping national information and communication technology (ICT) infrastructure to the requirements of potential digital health interventions in low- and middle-income countries
Source: J Glob Health. 2022 Dec 29;12:04094. doi: 10.7189/jogh.12.04094 (PMC9804211; doi:10.7189/jogh.12.04094)
Supplement: Online Supplementary Document [file jogh-12-04094-s001.zip › Appendix 2_RESPIRE news and online articles.docx]

**Mapping national Information and Communication Technology (ICT) infrastructure to the requirements of potential digital health interventions in low and middle income countries**

CY Hui, G M Monsur Habib, Parisa Khandakr, Chowdhury Zabir Hossain Tanim, Rutuja Patil, Ashish Satav, Shweta Panwar, Akshita Shukla, Animesh Nautiyal, Himanshi, Jitendra Nandkumar Shah, Mulya Nurmansyah, Fedri Ruluwedrata Rinawan, Adina Abdullah, Toh Teck Hock, Hani Salim, Zakiuddin Ahmed, Hana Mahmood, Dominique Balharry, Hilary Pinnock (correspondence: hilary.pinnock@ed.ac.uk)

**Appendix 2: RESPIRE news and online articles**

India

| **Topics** | **Types** | **Included in the review (highlighted)** | **Weblinks** |
| --- | --- | --- | --- |
| Health ID | News article | Digital health policy | 1. https://indianexpress.com/article/explained/ayushman-bharat-digital-mission-national-health-id-narendra-modi-7540456/ |
| National Guidelines for Data Quality in Surveys  Electricity quality access | News article | Electricity | 1. https://www.indiaspend.com/why-india-fails-to-supply-24x7-electricity-to-all-homes/ |
| Rural Internet users | News article | Electricity | 1. https://timesofindia.indiatimes.com/blogs/voices/beyond-access-the-future-of-electricity-in-india/ |
| Rural Internet users  Internet use as per Gender | News article | Statistics | 1. <https://www.thenewleam.com/2021/05/rural-internet-connectivity-in-india-gaps-and-challenges/> 2. [https://trai.gov.in/sites/default/files/Report_09112020_0.pdf] |
|  | News article | Statistics | 1. <https://feminisminindia.com/2020/09/28/infographic-indias-mobile-internet-gender-gap/> 2. [https://www.gsma.com/r/wp-content/uploads/2021/06/The-Mobile-Gender-Gap-Report-2021.pdf] |
| Internet use post COVID  Internet use post COVID | News article | Statistics | 1. <https://indianexpress.com/article/india/nfhs-data-shows-urban-rural-gender-gaps-in-internet-use-7103710/> 2. [Ministry of Health and Family Welfare. Government of India. National Family Health Survey - 5 2019-2021. http://rchiips.org/nfhs/NFHS-5_FCTS/India.pdf] |
|  | Literature | Strength and limitation | 1. https://www.ncbi.nlm.nih.gov/pmc/articles/PMC7280123/ |
|  | News article | Strength and limitation | 1. https://indiantelevision.com/television/tv-channels/viewership/tv-consumption-up-by-37-in-week-12-due-to-covid-19-lockdown-barc-india-nielsen-200402 |
| Sale surge in laptops post COVID | News article | Strength and limitation | 1. <https://www.business-standard.com/article/economy-policy/covid-19-lockdown-effect-every-second-indian-now-on-internet-in-cities-120050801805_1.html> |
| Sale surge in laptops post COVID | News article | Strength and limitation | 1. https://www.business-standard.com/article/companies/india-plugged-work-from-home-drives-laptop-sales-amid-covid-19-lockdown-120081101812_1.html |
| National Guidelines for Data Quality in Surveys  Electricity quality access | News article | Electricity | 1. https://www.indiaspend.com/why-india-fails-to-supply-24x7-electricity-to-all-homes/ |
| Rural Internet users | News article | Electricity | 1. https://timesofindia.indiatimes.com/blogs/voices/beyond-access-the-future-of-electricity-in-india/ |
| Internet use as per Gender | News article | Statistics | 1. <https://www.thenewleam.com/2021/05/rural-internet-connectivity-in-india-gaps-and-challenges/> 2. [https://trai.gov.in/sites/default/files/Report_09112020_0.pdf] |
|  | News article | Statistics | 1. <https://feminisminindia.com/2020/09/28/infographic-indias-mobile-internet-gender-gap/> 2. [https://www.gsma.com/r/wp-content/uploads/2021/06/The-Mobile-Gender-Gap-Report-2021.pdf] |
|  | News article | Statistics | 1. <https://indianexpress.com/article/india/nfhs-data-shows-urban-rural-gender-gaps-in-internet-use-7103710/> 2. [Ministry of Health and Family Welfare. Government of India. National Family Health Survey - 5 2019-2021. http://rchiips.org/nfhs/NFHS-5_FCTS/India.pdf] |
| Internet use post COVID | Literature | Strength and limitation | 1. https://www.ncbi.nlm.nih.gov/pmc/articles/PMC7280123/ |
|  | News article | Strength and limitation | 1. https://indiantelevision.com/television/tv-channels/viewership/tv-consumption-up-by-37-in-week-12-due-to-covid-19-lockdown-barc-india-nielsen-200402 |
| Sale surge in laptops post COVID | News article | Strength and limitation | 1. <https://www.business-standard.com/article/economy-policy/covid-19-lockdown-effect-every-second-indian-now-on-internet-in-cities-120050801805_1.html> |
| National Guidelines for Data Quality in Surveys  Electricity quality access | Survey report | Value (Background information) | 1. https://ndqf.in/wp-content/uploads/2021/07/National-Guidelines-for-DATA-QUALITY-in-Surveys.pdf |
| Information on the mobile network service provider | Government public information | Value  (Background information) | 1. https://www.trai.gov.in/consumer-info/telecom/service-provider-list |
| Most used Smartphones  Government portals on digital health | News article | Value  (Background information) | 1. https://www.reliancedigital.in/smart-phones/c/S101711?searchQuery=:relevance&page=0 |
|  | Government public information | Value  (Background information) | 1. https://ndhm.gov.in/ |
| Telemedicine practice guidelines | Government public information | Value  (Background information) | 1. https://esanjeevaniopd.in/Register |
| Telemedicine practice guidelines  Rural internet in India | Guideline | Value  (Background information) | 1. https://www.mohfw.gov.in/pdf/Telemedicine.pdf |
|  | News article | Value  (Background information) | 1. https://indianexpress.com/article/business/bharatnet-fails-to-enter-fast-lane-both-phases-staring-at-delays-amid-covid-7389075/ |
|  | News article | Value  (Background information) | 1. https://www.hindustantimes.com/business/govt-to-invite-bids-from-global-firms-for-rural-internet-project-101625102662619.html |
|  | News article | Value  (Background information) | 1. https://m.economictimes.com/industry/telecom/telecom-news/bharatnet-quality-poor-despite-payments-to-csc/articleshow/84485947.cms |
|  | News article | Value  (Background information) | 1. https://indianexpress.com/article/india/rural-net-project-it-ministry-dot-csc-7540423/ |
|  | News article | Value  (Background information) | 1. https://indianexpress.com/article/business/bharatnet-ppp-post-objections-on-bid-criteria-dot-to-issue-revised-tender-7584064/ |
| Air pollution  Air pollution | News article | Value  (Background information) | 1. https://timesofindia.indiatimes.com/business/india-business/intel-india-pilots-internet-over-power-lines/articleshow/88246959.cms |
|  | News article | Value  (Background information) | 1. https://timesofindia.indiatimes.com/city/mumbai/mumbai-low-cost-sensors-could-track-air-pollution-hotspots-says-study/articleshow/83652514.cms |
|  | News article | Value  (Background information) | 1. https://www.hindustantimes.com/india-news/who-tightens-air-quality-norms-over-health-hazard-101632339836591.html |
|  | News article | Value  (Background information) | 1. https://www.orfonline.org/expert-speak/taking-on-a-burning-problem-mumbais-air-pollution/ |
| Telecom service | News article | Value  (Background information) | 1. https://prana.cpcb.gov.in/#/partners |
| Telecom service | News article | Value  (Background information) | 1. https://mybs.in/2XA00HA |
| KEMHRC Vadu Study area Map | Map | Internet coverage and electricity (background information) | 1. https://www.kemhrcvadu.org/index.php/2014-03-02-11-17-47/vadu-hdss-maps |
| Census data on demography for Maharashtra village wise | Map | Internet coverage and electricity (background information) | 1. http://v3.communitygis.net/dashboard/health |

Malaysia

| **Topics** | **Types** | **Included in the review (highlighted)** | **Weblinks** |
| --- | --- | --- | --- |
| Low download speed | News article | Mobile signal | 1. <https://www-astroawani-com.translate.goog/berita-malaysia/kelajuan-muat-turun-kandungan-internet-di-malaysia-tidak-sampai-100-mbps-kajian-283039?_x_tr_sl=ms&_x_tr_tl=en&_x_tr_hl=en&_x_tr_pto=nui,sc> |
|  | Discussion paper | Mobile signal | 1. <http://www.krinstitute.org/assets/contentMS/img/template/editor/20210212%20Kualiti%20Jalur%20Lebar%20dan%20Teras%20Pembangunannya_v9.pdf> |
| Slow internet connection | News article | Mobile signal | 1. <https://bm.soyacincau.com/2021/01/21/laporan-ookla-speedtest-disember-2020-internet-malaysia-ketinggalan-asean/> |
| Increase internet use during the lockdown | News article | Strength and limitations | 1. http://www.krinstitute.org/assets/contentMS/img/template/editor/Views_Mutu%20Jalur%20Lebar%20Dalam%20Menghadapi%20Kejutan%20Covid-19_01.pdf |
|  | Youtube video | Strength and limitations | 1. https://www.youtube.com/watch?v=FXU3MJ2rwYk |
| Fragile ICT infrastructure | News article | Mobile signal | 1. https://medium.com/@kasprdata/slow-internet-how-covid-19-is-stressing-internet-infrastructure-in-your-country-f94ee6e3b156 |
| Poor internet connection in rural areas | News article | Mobile signal | 1. https://www-mcmc-gov-my.translate.goog/ms/media/press-clippings/skmm-proaktif-tangani-masalah-capaian-internet-sel?_x_tr_sl=ms&_x_tr_tl=en&_x_tr_hl=en&_x_tr_pto=nui,sc |
|  | News article | Mobile signal | 1. https://www.hmetro.com.my/mutakhir/2020/11/647378/berkhemah-di-atas-bukit-cari-line-nak-jawab-periksa-metrotv |
|  | News article | Mobile signal | 1. <https://www.hmetro.com.my/mutakhir/2020/12/651272/tahan-bau-najis-lembu-demi-liputan-internet-metrotv> |
| Slow internet connection | News article | Mobile signal (background information) | 1. <https://www.speedtest.net/insights/blog/tracking-covid-19-impact-global-internet-performance/?mc_cid=0dcbdb5bf2&mc_eid=e546476035#/Malaysia> |
| Fragile ICT infrastructure | News article | Mobile signal (background information) | 1. https://www.thestar.com.my/business/business-news/2020/01/09/lower-initial-5g-investments-in-targeted-sites |
| Mobile signal coverage map | Map | Mobile signal (background information) | 1. Maxis: (official website) <https://www.maxis.com.my/en/about-maxis/maxis-network/network-map/>   https://www.nperf.com/en/map/MY/-/10802.Maxis-Mobile/signal/?ll=5.00339434502215&lg=107.20458984375&zoom=6   1. Celcom: https://www.nperf.com/en/map/MY/-/82.Celcom/signal/?ll=4.12728532324537&lg=109.45678710937501&zoom=6 2. Official website - not so suitable for checking for overall coverage in Malaysia, provides more real time data https://www.celcom.com.my/support/network-checker 3. DiGi: (official website) http://coverage.digi.com.my:8080/fso_tsg/pages/VDServices.html   https://www.nperf.com/en/map/MY/-/5068.DiGi/signal/?ll=3.6230713262356864&lg=113.56018066406251&zoom=7   1. Unifi:(official website) https://unifi.com.my/personal/home/bebas-coverage#coverage   https://www.nperf.com/en/map/MY/-/44810.Unifi/signal/?ll=0.7909904981540058&lg=110.45654296875001&zoom=5   1. U Mobile: (official website) <https://www.u.com.my/support/selfhelp/coverage-map>   https://www.nperf.com/en/map/MY/-/5763.U-Mobile/signal/?ll=-0.2636709443366629&lg=107.457275390625&zoom=6   1. Yes 4G: (official website) <https://www.yes.my/support/coverage/> 2. https://www.nperf.com/en/map/MY/-/126418.Yes-4G/signal/?ll=3.995780512963038&lg=109.346923828125&zoom=6 |
| Electricity supply coverage map | Map | Electricity (background information) | 1. Malaysian Electricity Supply Statistical Information (by Suruhanjaya Tenaga, pg 76-79) 2. https://www.st.gov.my/en/contents/files/download/99/Performance_Statistical_Information_on_the_Malaysian_Electricity_Supply_Industry_2018.pdf |
| Electricity & gas supply infrastructure map | Map | Electricity (background information) | 1. https://www.st.gov.my/contents/2020/Maps/Electricity%20%26%20Gas%20Supply%20Infrastucture%20Malaysia%202019.pdf |
| Population map | Map | Mobile signal electricity (background information | 1. Population demographic statistics (First Quarter 2021) https://www.dosm.gov.my/v1/index.php?r=column/cthemeByCat&cat=430&bul_id=aVlJRDAvbjhWWEhQa1YvSWhsSjF3QT09&menu_id=L0pheU43NWJwRWVSZklWdzQ4TlhUUT09 |
| Current population estimates of the whole country | Government public information | Mobile signal electricity (background information | 1. https://www.dosm.gov.my/v1/index.php?r=column/cthemeByCat&cat=155&bul_id=OVByWjg5YkQ3MWFZRTN5bDJiaEVhZz09&menu_id=L0pheU43NWJwRWVSZklWdzQ4TlhUUT09 |
| Hospital and practice location map | Map | Mobile signal electricity (background information | 1. Public hospitals (MOH website): <https://www.moh.gov.my/index.php/database_stores/store_view/3?items=25&page=1> 2. Mapped locations: https://doi.org/10.1186/s40545-021-00308-9 |
| Spatial patterns of health clinics in Malaysia | Literature | Mobile signal electricity (background information | 1. https://www.researchgate.net/publication/271286644_Spatial_patterns_of_health_clinic_in_Malaysia |
| List of public health clinics (Klinik Kesihatan) in Malaysia | Government public information | Mobile signal electricity (background information | 1. https://www.moh.gov.my/index.php/database_stores/store_view/1?items=25&page=114 |
| List of public community clinics (Klinik Komuniti) in Malaysia | Government public information | Mobile signal electricity (background information | 1. https://www.moh.gov.my/index.php/database_stores/store_view/33?items=25&page=1 |
| Secondary care (in Malaysia) is offered in smaller public hospitals and more complex tertiary care, in regional and national hospitals (including university teaching hospitals run by the Ministry of Higher Education). | Literature (Systematic review) | Mobile signal electricity (background information | 1. https://apps.who.int/iris/handle/10665/206911 |

Indonesia

| **Topics** | **Types** |  | **Weblinks** |
| --- | --- | --- | --- |
| Regulation on data use | Regulations | Value  (Background information) | 1. 1 .Law Number 29 of 2004 concerning Medical Practice (Undang-Undang Nomor 29 tahun 2004 tentang Praktik Kedokteran) 2. 2. Law Number 44 of 2009 concerning Hospitals (Undang-Undang Nomor 44 Tahun 2009 tentang Rumah Sakit) 3. 3. Minister of Health Regulation No. 269/MENKES/PER/III/2008 Medical Records (Permenkes Nomor 269/MENKES/PER/III/2008 Rekam Medis) , https://ngada.org/menkes269-2008.htm 4. 4. Article 47 paragraph (3) of Law Number 29 of 2004 concerning Medical Practice (Pasal 47 ayat (3) Undang-Undang Nomor 29 tahun 2004 tentang Praktik Kedokteran) |

Bangladesh

| **Topics** | **Types** |  | **Weblinks** |
| --- | --- | --- | --- |
| Mobile network coverage by different operators | Map | Mobile signal (background information) | 1. https://www.nperf.com/en/map/BD/-/5900.Banglalink/signal/?ll=23.03929774776974&lg=91.18927001953125&zoom=6 |
| Access to electricity | Map | Electricity (background information) | 1. https://data.worldbank.org/indicator/EG.ELC.ACCS.UR.ZS?locations=BD&view=map |
| Bangladesh population map | Data | Electricity and mobile signal (background information) | 1. https://worldpopulationreview.com/countries/bangladesh-population |
