# Supplementary material for: Mapping national information and communication technology (ICT) infrastructure to the requirements of potential digital health interventions in low- and middle-income countries
Source: J Glob Health. 2022 Dec 29;12:04094. doi: 10.7189/jogh.12.04094 (PMC9804211; doi:10.7189/jogh.12.04094)
Supplement: Online Supplementary Document [file jogh-12-04094-s001.zip › Appendix 4_Other open sources data.docx]

Mapping national Information and Communication Technology (ICT) infrastructure to the requirements of potential digital health interventions in low and middle income countries

*CY Hui, G M Monsur Habib, Parisa Khandakr, Chowdhury Zabir Hossain Tanim, Rutuja Patil, Ashish Satav, Shweta Panwar, Akshita Shukla, Animesh Nautiyal, Himanshi, Jitendra Nandkumar Shah, Mulya Nurmansyah, Fedri Ruluwedrata Rinawan, Adina Abdullah, Toh Teck Hock, Hani Salim, Zakiuddin Ahmed, Hana Mahmood, Dominique Balharry, Hilary Pinnock (correspondence: hilary.pinnock@ed.ac.uk)*

**Appendix 4: Other open sources data**

**Percentage of urban and rural population who have access to electricity in the five countries (Source: The International Telecommunication Union [****^[[1]](#endnote-1)^])**

| Urban | 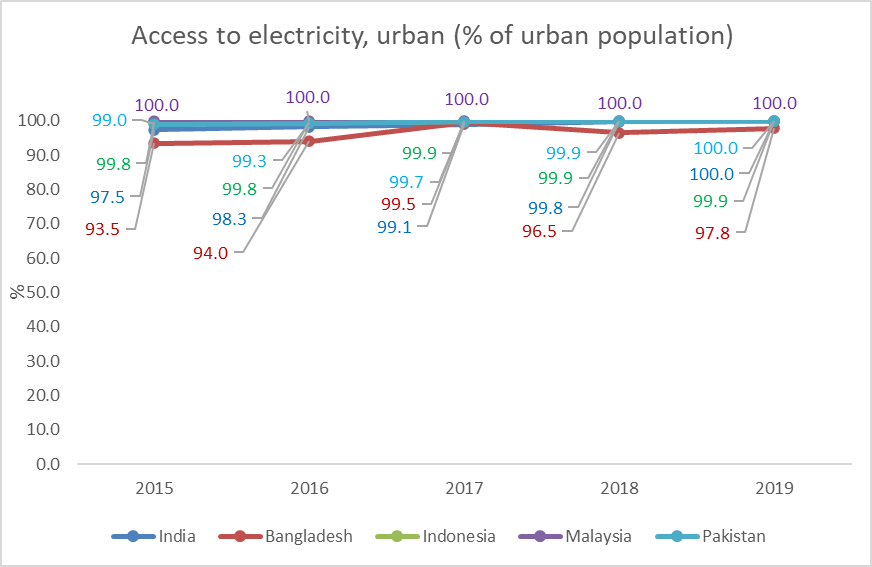 |
| --- | --- |
| Rural | 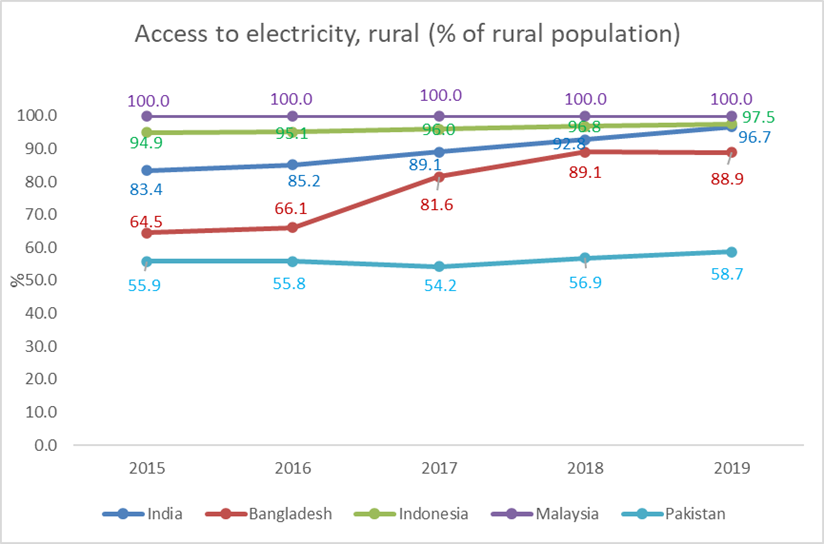 |

ICT prices and the affordability in 2020

(Source: The International Telecommunication Union[^[[2]](#endnote-2)^])

Note: Affordability line is defined as the 2 per cent of monthly Gross National Income (GNI) per capita, according to the UN Broadband Commission on Sustainable Development's Target 2 for 2025, ITU

| 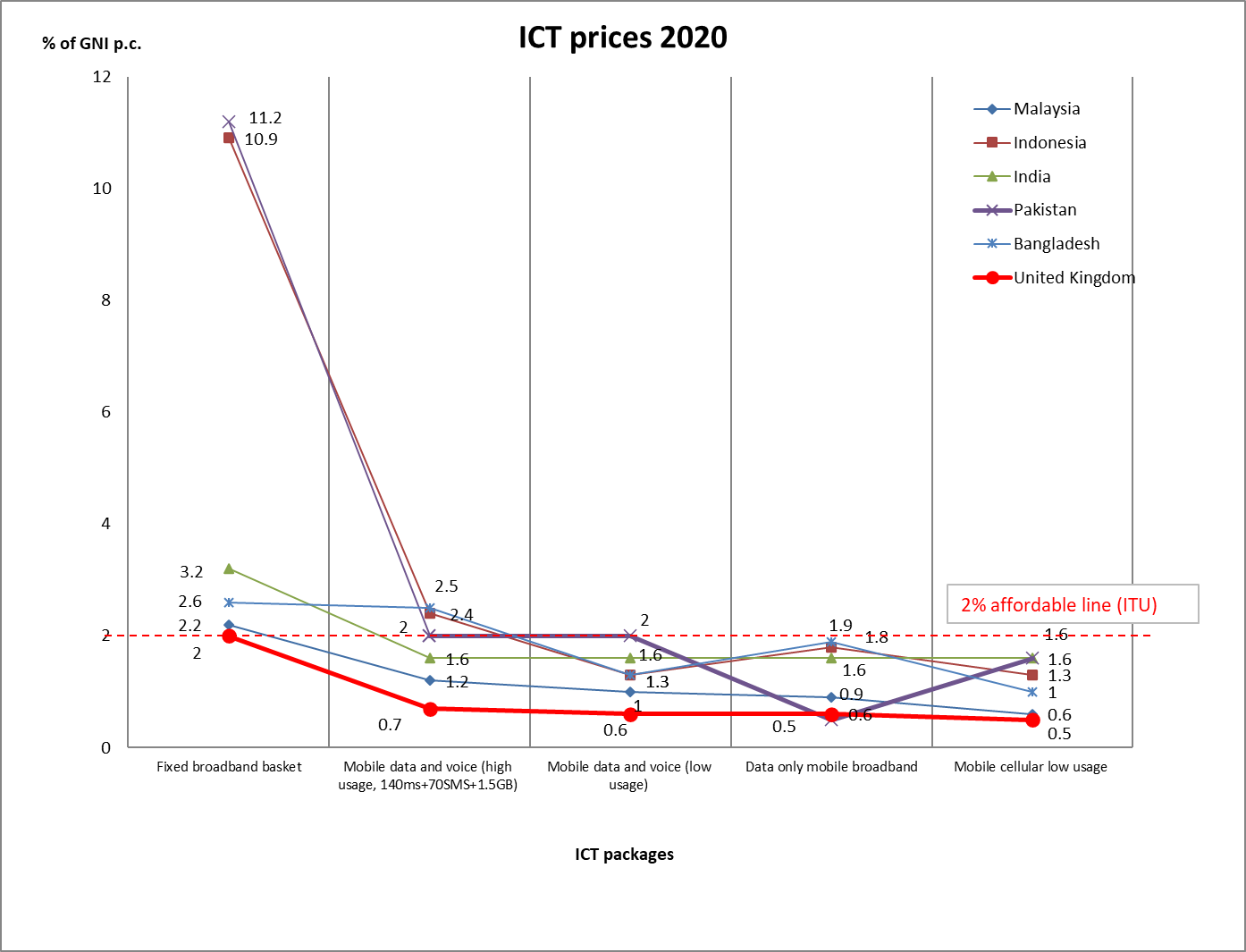 |
| --- |

The gender gap in the mobile ownership

(Sources: The International Telecommunication Union [1], Groupe Speciale Mobile Association. GSMA [^[[3]](#endnote-3)^])

Note: the gender gap was calculated as the (% of male-% of female)/% of male, defined by the GSMA connected woman


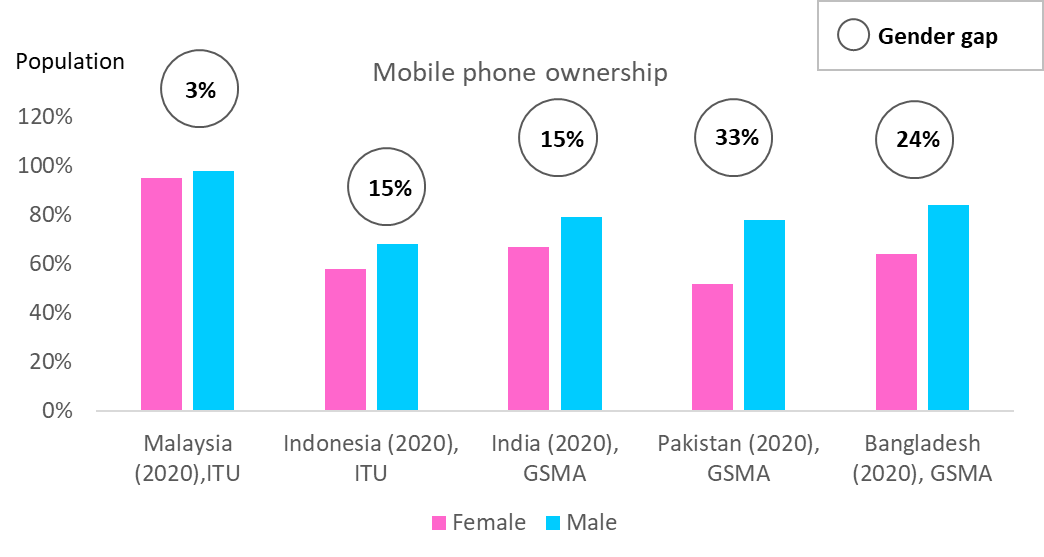


The gender gap in the use of internet

(Sources: The International Telecommunication Union [1], Ministry of Health and Family Welfare. Government of India[^[[4]](#endnote-4)^])

Note: the gender gap was calculated as the (% of male-% of female)/% of male, defined by the GSMA connected woman


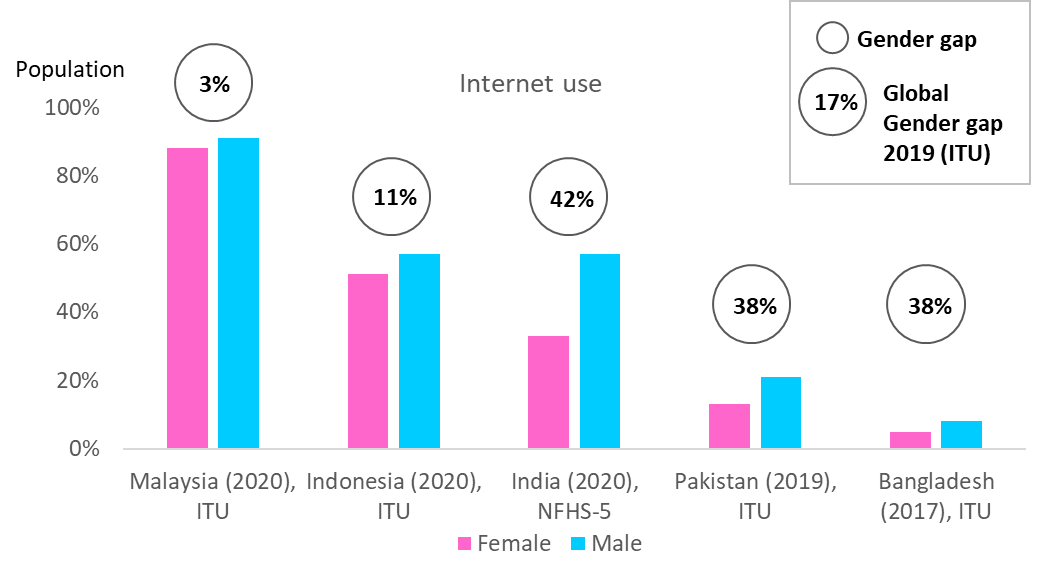


**References**

1. International Telecommunication Union(ITU). ICT Statistics. 2022. Available from https://www.itu.int/itu-d/sites/statistics/ (accessed date 23 June 2022) [↑](#endnote-ref-1)
2. International Telecommunication Union(ITU), Policy Brief - The Affordability Of Ict Services 2021. Available from [https://www.itu.int/en/ITU-D/Statistics/Pages/ICTprices/default.aspx (accessed date 23](https://www.itu.int/en/ITU-D/Statistics/Pages/ICTprices/default.aspx%20(accessed%20date%2023) June 2022) [↑](#endnote-ref-2)
3. Groupe Speciale Mobile Association. GSMA mobile gender gap report. 2021. Available from <https://www.gsma.com/r/wp-content/uploads/2021/06/The-Mobile-Gender-Gap-Report-2021.pdf> (accessed date 28 April 2022) [↑](#endnote-ref-3)
4. Ministry of Health and Family Welfare. Government of India. National Family Health Survey. 2019-2021. Available from http://rchiips.org/nfhs/NFHS-5_FCTS/India.pdf (accessed date 28 April 2022) [↑](#endnote-ref-4)
